# Editorial: Molecular basis of the energy management in cells: implications in health and disease

**DOI:** 10.3389/fmolb.2025.1578972

**Published:** 2025-03-12

**Authors:** Vanessa Checchetto, Simona Reina, Francesca Maria Garino, Marianna Flora Tomasello

**Affiliations:** ^1^ Department of Biology, University of Padova, Padova, Italy; ^2^ Department of Biomedical and Biotechnological Sciences, University of Catania, Catania, Italy; ^3^ Institute of Crystallography, National Council of Research, Catania Unit, Catania, Italy

**Keywords:** cellular bioenergetics, energy dysregulation, mitochondrial function, oxidative stress, Warburg effect, redox homeostasis, 21st-century diseases

Energy management in cells is a tightly regulated process critical for maintaining homeostasis, supporting survival, and responding to environmental changes at both the cellular and systemic levels. Central to this regulation lies cellular bioenergetics, the mechanisms by which cells generate, store, and utilize energy.

In current emerging research, energy dysregulation is identified as the common thread linking a diverse range of 21st-century diseases, including cancer, neurodegenerative disorders, diabetes, and cardiovascular conditions. One hypothesis for this energy crisis is that it stems from the rapid sociocultural evolution of human beings, which may have outpaced biological adaptation, leaving cells unable to appropriately balance energy production and detoxification of harmful by-products such as free radicals. The present Research Topic collects two reviews and three original papers exploring different aspects of energy use and regulation, either in cells or organisms. These papers also provide critical insights and new perspectives, as well as diagnostic/therapeutic innovations targeting energy regulating pathways.

The work by Naletova et al. reviews a fascinating paradigm of energy dysregulation, having potential adverse outcomes on reproductive biology, oncology and neurology, and offering potential biomarkers and therapeutic avenues to address metabolic crises across diseases. Authors first present GAPDS, a sperm-specific enzyme whose inactivation, under oxidative stress, disrupts ATP production thus resulting in male infertility. The author then proceed to examine GAPDS beyond its reproductive role. In melanoma cells, a truncated form of GAPDS exploits the glycolytic pathway to sustain tumor growth, amplifying the Warburg effect. Intriguingly, this same adaptation paradoxically inhibits metastasis, highlighting how energy reallocation introduces both strengths and vulnerabilities in cancer cells. Finally, the work considers the potential connection of GAPDH (the somatic GAPDS counterpart) with the development of neurodegenerative diseases. Oxidative modifications of GAPDH may promote amyloidogenesis, thereby linking energy failure to protein misfolding in conditions such as Alzheimer’s disease.

In the same vein, Brown’s work constitutes a critical re-evaluation of mitochondrial bioenergetics, thereby challenging long-standing assumptions concerning cellular energy dynamics. This innovative perspective is pertinent to a range of contexts, including neurodegeneration, cancer and ageing. Brown challenges the widely held view that mitochondria only produce heat when necessary, highlighting the crucial role of mitochondrial thermoregulation, particularly in the context of the brain, which already operates at a higher temperature than the rest of the body. With age, mitochondrial inefficiency may exacerbate heat-related protein misfolding, especially in the context of compromised vascularization and reduced reliance on glycolysis typical of neurodegenerative diseases such as Alzheimer’s. Brown also revisited the concept that glycolysis is less efficient than mitochondrial ATP production, emphasizing that, while producing less ATP, glycolysis is thermodynamically as efficient as mitochondria, whilst generating less heat. This adaptation may explain the Warburg effect in cancer, where aerobic glycolysis is preferred to reduce heat buildup in poorly vascularized tumors. Similarly, glycolysis minimizes heat stress in exercising muscles, underscoring its role in pathological and adaptive responses. Furthermore, the prevailing notion of mitochondria being the predominant source of reactive oxygen species (ROS) is addressed. Brown points out as other organelles, such as peroxisomes and the endoplasmic reticulum, often surpass mitochondria in ROS production, contributing to tumorigenesis, metastasis, and neurodegeneration when antioxidant defences are overwhelmed. By looking at these bioenergetic concepts under a different light, Brown posits an atypical understanding of mitochondria playing central roles in terms of heat production, energy regulation and metabolic flexibility.

Similar to the review by Naletova et al., and the perspective by Brown, in their original research article, Cilenti et al., examines mitochondrial bioenergetics and cross-disease mechanisms. The study focuses on the mitochondrial E3 ubiquitin ligase MUL1, which plays an essential role in mitophagy and lipid metabolism. The authors demonstrate that despite defective mitophagy, MUL1 deficiency reduces the high-fat diet (HFD)-induced obesity, hepatic steatosis, insulin resistance, and glucose intolerance in mice. By using lipidomic and transcriptomic analyses they report reduced triglyceride levels, altered lipid storage, and an upregulation of Complex I subunits, which collectively mitigate the effects of HFD. This study underscores the distinctive function of MUL1 in mitochondrial quality control and underscores the translational potential of targeting mitochondrial pathways in the treatment of metabolic disorders, such as obesity.

Fanelli et al. analyzed alterations in metabolic and oxidative stress in SAOS-2 osteosarcoma cells under mechanical stimulation, thereby providing insights into the impact of mechanical forces on tumor behavior. Mechano-transduction has been demonstrated to play a pivotal role in the progression of tumors by exerting its influence on cancer metabolism and stress responses. This study demonstrates that cyclic uniaxial stretch exerts an effect on glycolysis, the TCA cycle and amino acid metabolism in SAOS-2 cells, thus establishing a link between mechanical stress and metabolic adaptation. While osteosarcoma cells typically favor enhanced glycolysis, mechanical stress has been shown to downregulate glycolysis and the TCA cycle, while enhancing glutaminolysis, leading to glutathione depletion, ROS accumulation and increased oxidative stress.

The findings highlight the potential of mechanobiological interventions in regulating redox homeostasis, increasing cell sensitivity to oxidative stress-based therapies. It is also notable that mechanical stimulation enhances doxorubicin-induced toxicity, as evidenced by the disruption of cytoprotective gene expression, further sensitizing osteosarcoma cells to chemotherapy. These results position mechanotherapy as a promising approach.

This final contribution, by Vekaria et al., provides a valuable innovative tool enabling mitochondrial, ETC., complex I/II/IV flux analysis in frozen homogenates/isolates. The authors developed a fluorescent-based Seahorse assay to evaluate traumatic brain injury (TBI) effects in permeabilized mitochondria supplemented with cytochrome c. While incapable of assessing membrane potential or TCA flux, the method detected injury-specific deficits in ETC complexes within fresh-tissue-derived mitochondria/homogenates stored at −80°C, unlike mitochondria isolated from frozen whole tissues. Requiring minimal sample, it identifies subtle dysfunctions and is adaptable to neurodegenerative disease models, offering a resourceful solution for studying mitochondrial pathophysiology in stored samples.

In summary, the contributions included in this concise Research Topic all offer hints of improving our understanding of the origins of various diseases and inspire innovative strategies to address the global health burden caused by the failure of energy homeostasis.

